# Intravitreal Combination of Dexamethasone Sodium Phosphate and Bevacizumab in The Treatment of Exudative AMD

**DOI:** 10.1038/srep08627

**Published:** 2015-02-27

**Authors:** N. Vakalis, G. Echiadis, A. Pervena, I. Deligiannis, E. Kavalarakis, S. Giannikakis, I. Papaefthymiou

**Affiliations:** 1 Consultant in ophthalmology, Department of Ophthalmology, Naval Hospital of Athens, Greece; 2 Resident in ophthalmology, Department of Ophthalmology, Naval Hospital of Athens, Greece; 3 Consultant in ophthalmology, Head of Department of Ophthalmology, Naval Hospital of Athens, Greece

## Abstract

The purpose of this study is to investigate the efficacy and safety of intravitreal dexamethasone sodium phosphate (DSP) combined with bevacizumab for the treatment of neovascular age-related macular degeneration (AMD). In this non comparative case study, 30 eyes of 27 patients with CNV due to AMD received intravitreal DSP (0.2 mg) and bevacizumab (1.25 mg) during a 6-month PRN (pro re nata) dosing regimen. Visual acuity, macular thickness and intraocular pressure (IOP) were monitored and recorded. After 6 months, mean retinal thickness decreased from 423.5 ± 75.3 to 228.2 ± 34.5 and mean visual acuity improved from 0.9 ± 0.39 logMAR to 0.53 ± 0.34 (p = 0.001) logMAR. During the trial period, 81 intravitreal injections were performed in 30 eyes, thus the mean number of injections per eye was 2.7 ± 1.1. 86.7% of the eyes required 3 or less injections while only 13.3% needed 4 or more injections. None of the patients, phakic or pseudophakic, manifested an elevation of IOP during the treatment, ranging between 12 and 22 mmHg. Combined DSP and bevacizumab offers encouraging results in the challenge of AMD treatment, providing immediate response of macular edema, reduced number of intravitreal injections and stabilization or improvement of visual acuity.

Age-related macular degeneration (AMD) constitutes the main cause of irreversible vision loss in the elderly (>50 years) in the western world and the third leading cause of blindness worldwide^1^,^2^. Exudative AMD is the most severe form of the disease and is considered responsible for the majority of significant vision loss in AMD^3^,^4^. In this form of the disease, a choroidal neovascular membrane (CNV) formation beneath the macula causes leakage with macular hemorrhage and serous fluid, which leads to vision impairment. Recent studies of intravitreal anti-VEGF treatment (ranibizumab, bevacizumab and aflibercept) have been very promising in the treatment of neovascular AMD.

Vascular endothelial growth factor (VEGF) has a key role in the pathogenesis of exudative AMD and in ocular neovascularization in general^5^. It is responsible for increased vascular permeability while it also promotes endothelial cell survival. Furthermore, it is considered a chemotactic factor for leucocytes. The aforementioned chemical properties of VEGF are responsible for triggering angiogenesis and stimulating CNV membrane formation^5^. Bevacizumab is a full-length monoclonal antibody that binds all isoforms of VEGF. Off-label intravitreal injections of bevacizumab for neovascular AMD was first documented in 2005^6^. The therapeutic use of corticosteroids for inflammatory eye diseases was first described in 1951^7^.

The cardinal effects of steroids are considered to be stabilization of the blood-retinal barrier (BRB), reduction of exudation, and downregulation of inflammatory agents. Studies in rabbit models suggest that corticosteroid administration blocks the VEGF-induced blood-retinal barrier breakdown and subsequent intraretinal edema by modulating the signaling and downregulation of the effector proteins acting on the VEGF receptor^8^.

Current scientific hypothesis suggests that the combination of intravitreal bevacizumab and dexamethasone sodium phosphate (DSP) can reduce the recurrence rate after initial treatment and increase the time between subsequent intravitreal injections^9^. Several studies concluded that the use of DSP with verteporfin photodynamic therapy (PDT) and anti-VEGF agents can decrease the number of required anti-VEGF injections and stabilize or improve visual acuity in neovascular AMD patients^10^,^11^,^12^.

## Purpose

The aim of the present study is to investigate the efficacy and safety of intravitreal DSP (0.2 mg) and bevacizumab (1.25 mg) for the treatment of neovascular AMD during a 6-month PRN dosing regimen. This is a pilot study intended to monitor the recurrence rate, the macular thickness and the visual acuity of patients with neovascular AMD.

## Methods

The study protocol (Study N.:10 rec no24, November 4,2013) was approved by the scientific and research ethics committee of Naval Hospital of Athens (Prot. N. 11/13, November 8, 2013). Informed consent for both the treatment and participation in the research was obtained from each patient and the research was conducted according to the provisions of the Declaration of Helsinki.

A total of 30 eyes who met the inclusion criteria were enrolled. These criteria included age >50 years, presence of neovascular AMD, no use of systemic steroids, no previous treatment for AMD or inflammatory CNV as presumed ocular histoplasmosis syndrome (POHS) and punctuate inner choroidopathy (PIC) (Negative history, clear fundus without lesions or scars, confirmed by FFA), no ocular history of glaucoma, diabetic retinopathy or previous pars plana vitrectomy and no medical history of recent cerebral or myocardial infarction.

Thirty eyes in total were treated with 1.25 mg intravitreal bevacizumab and 0.2 mg DSP. The following baseline (BSL) data were recorded at the initial visit and at each subsequent follow-up visit: Best-corrected visual acuity (BCVA) using a 6-m Snellen chart, intraocular pressure (IOP), slit-lamp and fundus examination and central macular thickness via optical coherence tomography (OCT). In addition, fundus fluorescein angiography (FFA) was performed at the initial visit.

After discussion with the patient regarding the risks, benefits, alternatives to treatment and the off-label use of bevacizumab, informed consent was obtained for the use of intravitreal bevacizumab combined with DSP. Intravitreal bevacizumab (1.25 mg [0.05 ml]) and DSP (0.2 mg [0.05 ml]) was injected via the pars plana using a 30-gauge needle. The procedure was performed in the operating room under sterile conditions. The patient received an initial drop of lidocaine 4% onto the study eye followed by 5% povidone-iodine solution 1 minute prior to the injection. The eyelid margins, the eyelids and the periocular skin were then washed carefully with povidone-iodine. The eye was draped in a sterile way. A sterile lid speculum was inserted by the surgeon. Another drop of 5% povidone-iodine was applied onto the eye. A caliper was used to mark the injection site 3.5 mm from the limbus in pseudophakic eyes and 4.0 mm in phakic eyes in the inferior temporal quadrant. Ten international units of bevacizumab and 10 international units of DSP were drawn into an insulin syringe. From the combined mixture, a total of 0.1 ml was slowly injected at the marked site at a 90 degree angle. The needle was then slowly withdrawn and a drop of 5% povidone-iodine applied. Ten seconds massage of the globe was performed and the patient was examined via indirect ophthalmoscope for spontaneous retinal venous pulse confirmation.

Post procedure antibiotic drops were prescribed qid for 4 days. Patients were followed up at days 2, 6, 15, 30 and then at 1-month intervals. Additional injections were performed in the event of macular thickness >250 μm or in presence of recurrence. Recurrence was defined as an increase of macular edema, subretinal or intraretinal fluid as determined by OCT or by the presence of macular hemorrhage via slit lamp examination. Best corrected visual acuity (BCVA) was measured using spectacles and/or pin-hole on a Snellen chart and converted to logarithm of the minimum angle of resolution (logMAR) units for the purpose of data analyses. Central macular thickness (CMT) was assessed using OCT scan (Stratus III OCT, Carl Zeiss).

### Statistical Methods

Summary statistics of all continuous variables were based on measures of central tendency and dispersity (mean + sd) whereas categorical variables were described via tables of relative and absolute frequencies. The statistical evaluation of change over time regarding the macular thickness and the visual acuity was assessed by mixed models of repeated measures.

Kaplan-Meier product limit curve was employed to illustrate the disease free distribution curve of the sample throughout the 6 month observation period. The median time to relapse was provided. The influence of the demographic and clinical characteristics (age, sex, phakic or pseudophakic state and baseline macular edema) to the disease free distribution were evaluated by Cox's proportional hazard regression model.

## Results

In this pilot study, 30 eyes of 27 patients (19 men, 8 women) that met the eligibility criteria were included. The mean age of patients was 77.4 years (range 54–91, SD 7.6). Twenty eyes were pseudophakic, 7 were phakic and 3 had cataract before study entry and underwent phacoemulsification surgery during the 6 month period of treatment. None of these patients had received previous treatment for the disease. All patients received an intravitreal injection at day 0 and assessed at days 2, 6, 15, 30 and then at 1 month intervals. Additional injections were performed in two cases: (A) During the improvement, when the expected results (macular thickness <250 μm) were not met and (B) in case of recurrence of the disease (increased macular thickness >250 μm).

70% of the eyes (21 eyes) had at least 1 episode of recurrence, while 30% (9 eyes) did not relapse at all during these 6 months. Furthermore, the median time to relapse was 4 months and it did not depend on age, sex, phakic or pseudophakic state and macular edema at baseline.

During the study period of 6 months, 81 intravitreal injections were performed in 30 eyes, thus the mean number of injections per eye was 2.7 ± 1.1. 86.7% of the eyes required 3 or less injections while only 13.3% needed 4 or more injections during the 6 month period (Table 1).

**Figure 1:**
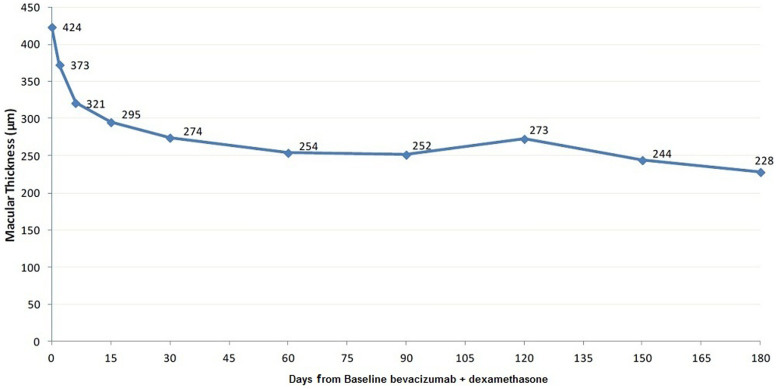
Mean macular thickness in time.

When assessing the entire population, macular thickness is significantly decreased over the period of six months as compared to baseline. Patient response to treatment was immediate. Just 2 days after the first injection the mean macular thickness was reduced by 11.7 ± 5.9%, while 6 and 15 days later, the reduction from baseline was 24 ± 9.8% and 29.6 ± 11.4% respectively. Baseline mean macular thickness was 423.5 ± 75.3 μm while at 3 and 6 months was 251.5 ± 67 μm and 228.2 ± 34.5 μm respectively (Figure 1, Table 2).

In Table 3, the relative reduction of macular thickness in non relapsed patients is reported. At the first month follow-up visit, 3 patients relapsed and thus they were excluded from the “1 month” horizontal row, while another 6 relapsed at the second month visit and were excluded in the “2 months” row and so on, therefore it is evident that most of the macular thickness reduction occurred in the first month.

**Figure 2:**
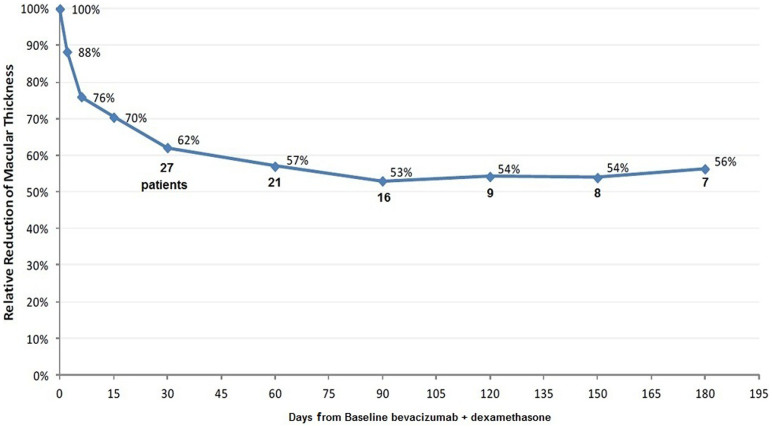
Relative reduction of macular thickness in non relapsed patients.

The changes that were recorded in the following months were less significant and a “plateau phase” took place, 30 days after the introduction of the treatment and until the end of the study (Figure 2). Consequently, the evaluations of the first month results after baseline are highly predictive for the long-term outcome.

**Figure 3:**
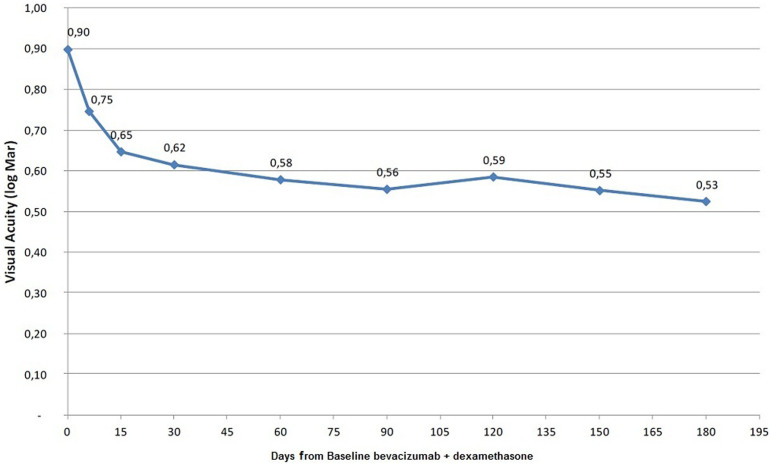
Best corrected visual acuity in time.

Visual acuity also improved significantly from baseline 6/48 Snellen (equivalent 0.9 ± 0.39 logMAR) to 6/24 Snellen (equivalent 0.62 ± 0.34 logMAR) after 1 month. After the first month, there is a “plateau phase”, similarly to the macular thickness graph, concluding that most of the beneficial effect of the treatment occurs within 1 month and followed by less significant changes (Figure 3). Final mean BCVA at 6 months improved to 6/19 Snellen, logMAR equivalent 0.53 ± 0.34 (P = 0.001).

## Discussion

The main cause of VEGF induction in various cell types appears to be hypoxia^13^,^14^,^15^,^16^,^17^. Hypoxia promotes the expression of VEGF and HIF-1α in mice lung tissue. DSP inhibits the expression of VEGF and HIF-1α of hypoxic mice and thus it is capable of suppressing the angiogenesis^18^. The retina is a tissue of high oxygen demand and thus susceptible to oxidative stress: Cellular damage mediated by reactive oxygen intermediates (ROI)^19^. Inflammation is implicated in both severe dry AMD with atrophy and choroidal neovascularization.

Increased inflammation is closely associated with changes in the complement system and the progression of AMD. Various complement components, such as C3, C5, C5b-9, CFH have been identified in drusen and AMD lesions. Furthermore drusen contain a series of inflammatory molecules incuding vitronectin, amyloid A/P, factor X, prothrombin and in some instances, immunoglobulin, HLA-DR and CRP, suggesting a strong correlation between chronic inflammation and AMD^20^,^21^.

Macrophages are identified in proximity of the RPE cells in animal models of experimental autoimmune uveitis^22^,^23^ and in a series of ocular inflammatory conditions such as proliferative vitreoretinopathy and CNVM^24^,^25^.

The production of angiogenic mediators such as TNF-a, VEGF, IL-1, bFGF and TGF- β^26^,^27^,^28^ implicates macrophages in neo-angiogenesis. Another study demonstrated that IL-1b and TNF-a secreted by macrophages promotes neo-angiogenesis by triggering the RPE cells in producing VEGF^29^. Further research confirmed that monocyte chemotaxis induces neovascularization^30^ and correlates macrophage accumulation with the extent of the angiogenesis after vascular occlusion^31^.

AMD pathogenesis is multifactorial and consists of a vicious circle which involves hypoxia, oxidative stress, inflammation, edema and finally neovascularization. While anti-VEGF agents achieve their therapeutic effect only through VEGF inhibition, corticosteroids have a more wide therapeutic mechanism with their anti-inflammatory, anti-exudative, and anti-angiogenic properties. Furthermore, they prevent blood-retinal barrier breakdown^8^.

The combination of anti-VEGF and corticosteroids has proved its efficiency in recent AMD studies. In particular, it has been shown that intravitreal combination of triamcinolone and bevacizumab is highly efficient in decreasing the amount of subretinal fluid, limiting neovascularization and preserving or increasing visual acuity^32^. Similar positive results were obtained in a recent study of combined intravitreal ranibizumab and dexamethasone^33^.

In this study, DSP was preferred over triamcinolone because of its high anti-inflammatory potency (Six times higher potency than triamcinolone)^34^, fast bioavailability, transparency and immediate action. DSP can also access the posterior retina, from the vitreous through the retina. This characteristic makes it highly efficient in posterior eye disease therapy^35^.

Furthermore an in vitro study demonstrated that the association of bevacizumab with DSP causes stabilization of the antibody in comparison with the sample of bevacizumab alone^36^.

Intravitreal DSP in doses up to 0.8 mg has been used for treating endophthalmitis and as complementary treatment during vitrectomy in diabetic patients, without any relevant ocular toxicity^37^. The DSP dose in this study was 0.2 mg.

The most common adverse effect of corticosteroid intravitreal injection is undoubtedly the increase of IOP. None of the patients, phakic or pseudophakic, in this study manifested an elevation of IOP during the treatment, ranging between 12 and 22 mmHg (Measured in day 2, 15, 30 and in 1 month intervals). This could be attributed to the rapid action, short duration and short-time clearance of dexamethasone in the vitreous^38^.

In the current study none of the phakic patients presented a development or deterioration of pre-existing cataract. The grading of cataract was evaluated with the WHO/PBD simplified cataract grading system. The task was assigned to a unique ophthalmologist in order to ensure the minimum amount of subjectivity. LOC III grading system was not preferred because of the fact that the cataract was not the main target of this study. None of the patients presented ocular or systemic manifestations, attributable to the intravitreal injection itself, or the substances implicated, such as vitreous hemorrhage, retinal tear or detachment, allergy, cataract (from inadvertently hitting the lens) and infection (endophthalmitis).

Ten seconds massage of the globe and confirmation of optic nerve venous pulse were performed as a safety measure for the rapid IOP raise due to the increased intraocular volume after the injection. None of the study eyes presented a non perfusion optic nerve head. Additionally, the advantages of DSP in comparison to triamcinolone and anti-VEGF substances are the very low cost, the use of 30 G injection needle and the absence of IOP increase.

**Figure 4:**
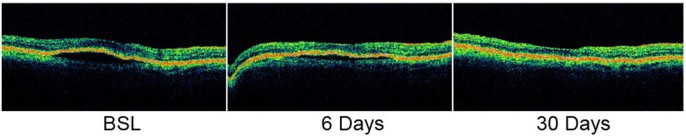
Case of PED decrease.

Five patients of the study displayed pigment epithelial detachments (PEDs) at first visit. Four of them improved with a decrease of PED. However, the quantitative evaluation of this change could not be measured with the current OCT Stratus software since it measures the distance between the RPE layer and the ILM and thus, does not include the PED thickness in the calculations (Figure 4).

The data presented in this study demonstrate that a significant reduction of macular edema, accompanied by visual acuity improvement, occurs rapidly in all patients treated with combined DSP and bevacizumab intravitreal injections. All patients displayed reduced macular thickness and improved BCVA even after thirty-six to forty-eight hours (36–48 hours). Six days after the first injection the mean macular thickness of the entire population of the study was reduced by 24 ± 9.8%, while BCVA improved from log MAR 0,9 ± 0,39 to log MAR 0,75 ± 0,38.

**Figure 5:**
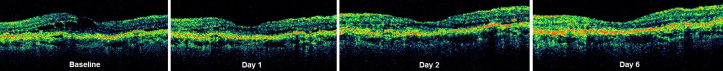
Case of macular edema rapid decrease.

Quoting such a case, a 76 year old woman presented with exudative AMD in the right eye, with subretinal fluid and 360 μm of macular thickness. After combined treatment with DSP and bevacizumab, macular thickness was reduced to 190 μm and 160 μm after 1 and 2 days respectively, while after 6 days the macular thickness was furtherly reduced to 135 μm (Figure 5). BCVA, using the Snellen visual acuity chart, was improved from 3/10 to 4/10 after 2 days and 6/10 after 6 days.

We noticed that patients with a history of hypertension, smoking and hypercholesterolemia were more prone to relapses and less responsive to the treatment, when compared to patients without the aforementioned risk factors. We are planning to further investigate these findings in the future.

Encouraging case report announcements of intravitreal dexamethasone in diabetic macular edema (DME)^39^ and retinal vein occlusion (RVO)^40^ treatment and combined bevacizumab and dexamethasone for the treatment of exudative AMD^41^, motivated the clinic to take one step forward and proceed with this study.

The treatment of exudative AMD is not limited to 6 months. More data and longer periods of monitoring are necessary to determine the efficacy, safety and injection frequency of intravitreal DSP and bevacizumab combination. A comparative study between bevacizumab “preloaded” (3 monthly injections initially) and bevacizumab combined with dexamethasone PRN dosing regimens is currently in progress. Nevertheless, a flexible watch-and-wait strategy appears feasible and continuous careful monitoring is of pivotal importance in preventing the recurrence of visual loss.

## Conclusions

Various studies have demonstrated that VEGF and inflammatory cells are acting in an interactive way in the pathogenesis of neovascular AMD. Consequently, a new approach in the treatment of AMD which includes the inhibition of VEGF and the reduction of inflammation should be put under consideration^42^,^43^. This study, focusing on these targets by using DSP and bevacizumab, offers encouraging results in the challenge of AMD treatment, providing immediate response of macular edema, reduced number of intravitreal injections and stabilization or improvement of visual acuity.

## Author Contributions

V.N. conceived and designed the study. E.G. and P.A. wrote the main manuscript text. D.I., G.S. and K.E. prepared tables 1–3 and figures 1–5. E.G., P.A. and G.S. collected patient data. P.I. edited the manuscript. All authors reviewed the manuscript.

## Figures and Tables

**Table 1:** Total No of Injections at 6 months

	Frequency	Percent
1 inj	4 eyes	13.3
2 inj	8 eyes	26.7
3 inj	14 eyes	46.7
4 inj	1 eye	3.3
5 inj	3 eyes	10.0
Total	30	100.0

**Table 2:** Mean macular thickness in time

	Ν	Mean	SD	Min	Max	p-value
**Day 0**	30	423.5	75.3	300	590	Ref cat
**Day 2**	30	372.7	64.3	255	520	<0.001
**Day 6**	30	321.2	64.7	170	495	<0.001
**Day 15**	30	295.3	58.6	170	410	<0.001
**1 month**	30	274.4	76.0	160	500	<0.001
**2 months**	30	254.0	42.5	180	360	<0.001
**3 months**	30	251.5	67.0	160	430	<0.001
**4 months**	30	272.8	91.2	150	550	<0.001
**5 months**	30	244.2	64.4	140	420	<0.001
**6 months**	30	228.2	34.5	135	290	<0.001

**Table 3:** Relative reduction of macular thickness in time in non relapsed patients

	N	Mean	SD	Min	Max	p-value
**BSL**	30	100%				Ref cat
**Day 2**	30	88.3%	5.9%	69.4%	95.6%	<0.001
**Day 6**	30	76.0%	9.8%	54.8%	90.9%	<0.001
**Day 15**	30	70.4%	11.4%	43.9%	86.7%	<0.001
**1 month**	27	62.1%	12.1%	37.7%	82.5%	<0.001
**2 months**	21	57.0%	13.3%	37.9%	81.7%	<0.001
**3 months**	16	53.0%	12.1%	30.4%	73.5%	<0.001
**4 months**	9	54.2%	10.5%	36.0%	68.5%	<0.001
**5 months**	8	53.9%	12.1%	36.0%	69.9%	<0.001
**6 months**	7	56.3%	10.9%	40.9%	69.9%	<0.001
